# The cost of living crisis – how does it impact the health and life of individuals? A survey exploring perceptions in Italy, Germany, Sweden and the United Kingdom

**DOI:** 10.1186/s12889-024-19330-y

**Published:** 2024-07-09

**Authors:** Kate Grailey, Roberto Fernandez-Crespo, Peter Howitt, Melanie Leis, Ara Darzi, Ana Luisa Neves

**Affiliations:** 1 Centre for Health Policy, Institute of Global Health Innovation, Imperial College London, Room 1035, 10th Floor QEQM, St Mary’s Campus, W2 1NY London, U.K.; 2 Department of Primary Care and Public Health, Imperial College London, London, U.K.; 3 NIHR North West London Patient Safety Research Collaboration, Institute of Global Health Innovation, Imperial College London, London, U.K.

**Keywords:** Cost of living crisis, Healthcare, Health policy, Inequity

## Abstract

**Background:**

The Cost of Living Crisis (CoLC), a real term reduction in basic income, risks individuals being unable to afford essentials such as heat, food and clothing. The impact of the CoLC is disproportionate – with different population sub-groups more likely to be negatively affected. The objective of this survey was to evaluate the perceived impact of the CoLC on the life and health of participants across four European countries.

**Methods:**

A survey housing two questions to investigate the relationship between the CoLC and its perceived impact on life and health was developed. Four European countries (U.K., Sweden, Italy and Germany) took part via the YouGov platform. Logistic regression models were created for each country and question to evaluate which population characteristics were associated with a negative reported impact of the CoLC.

**Results:**

A total of 8,152 unique individuals responded between 17th March and 30th March 2023. Each country was equally represented. Those aged 36–64 were more likely to report a negative impact of the CoLC on their life and health than younger participants (*p* < 0.001, *p* = 0.02 respectively). Across all countries, females were significantly more likely to report a negative impact on their life and health, however, when analysed according to country, in Sweden females were less likely to report a negative impact (*p* < 0.001). Those in lower income families or who reported poor health in the preceding 12 months were significantly more likely to report a negative impact of the CoLC on their life and health. There was no difference within the participant group on the reported impact of the CoLC based on location (rural vs. urban).

**Conclusions:**

We demonstrate the disproportionate negative impact of the CoLC on both life and health in different population subgroups. Germany and Sweden appeared to be more resilient to the effects of the CoLC, particularly for certain population subgroups. It is important to understand the differing effects of a CoLC, and to learn from successful health and economic strategies in order to create targeted policy and create a population resilient to economic shocks.

**Supplementary Information:**

The online version contains supplementary material available at 10.1186/s12889-024-19330-y.

## Introduction

The cost-of-living crisis (CoLC), defined by homeless charity Crisis as “*a period of time during which the cost of everyday essentials like food and bills increases more quickly than average household income*” [1] has led to a reduction in an individual’s “real” income, leading to millions of people across the world struggling to meet the costs of basic needs. The United Kingdom has been reported as being in a CoLC since late 2021 [2], with similar economic situations mirrored worldwide [3].

Since 2021, inflation has risen globally. Between quarter 4 (Q4) of 2020 and Q4 of 2022, the U.K. saw inflation rise from 0.8 to 9.4%. Italy suffered the most considerable increase in inflation of major economies, reaching 12% by Q4 2022. Food prices in Italy rose by 11.6% across 2022, with minimal wage increase (just 1% in 2022) [4, 5].

This situation is mirrored across Europe and is having a demonstrable impact on the lives of citizens, even in countries such as Sweden (with an inflation rate of around 8% in Q4 2022) who typically have more robust welfare provisions [6, 7]. Even though poverty rates in Sweden are lower than the European average, citizens are still suffering with doubling energy prices, with the price of some food items rising by up to 25% [6].

In Germany, 11.8% of the population spent more than 40% of their disposable household income on housing costs in 2022, paying up to three times the price for gas [8]. By the end of 2022, the rate of inflation was 8.6% [9]. Whilst this is lower than some other European countries, it remains substantially higher than the European Union target of 2%. In 2023, the cost-of-living index ranked the U.K. 33rd of 140 countries, with Italy ranking 35 and Germany at 30 [5].

The aetiology of the current CoLC is multifactorial – with increasing taxes, rising energy prices, and stagnant wages all contributing. The crisis is additionally being driven in varying degrees by external events such as the COVID-19 pandemic and the Ukraine-Russia war [10]. In particular, it is postulated that the current CoLC has had greater impact on those living in poverty or on lower incomes as a consequence of it arising so close after the COVID-19 pandemic and its associated economic impacts [11], and there is a risk that this CoLC will push households and individuals experiencing financial hardship prior to the crisis into poverty [12]. Whilst rising costs affect all socioeconomic groups – it is likely that those on lower incomes will be disproportionately affected, as a larger proportion of their income is spent on household “essentials” such as food and heating [10]. We know that there are rising numbers of individuals affected by food insecurity [13] and increasing prevalence of psychological distress (using the increase in consumption of anti-depressant medications as a proxy measure) [14]. There is inequity in the impact of this economic crisis – with those in more vulnerable groups being disproportionately impacted and at higher risk of worsening health [15].

It is not difficult to correlate that challenges in affording the essentials required to simply exist (heating, water, food) will have a significant impact on health and wellbeing, including cold-related mortality, malnutrition, and worsening mental health [12, 16]. This may be particularly marked for certain population subgroups including those with pre-existing health conditions, such as cancer [17]. This impact may be explained due to the effect of the CoLC on health behaviours (e.g., change in dietary intake), material changes (e.g., housing insecurity or inability to afford medications), or psychosocial effects (e.g., strain on personal relationships or reduced social activities) [12]. Other publications have outlined the potential issues this crisis may have on children’s health and wellbeing [18, 19] and obesity [20].

There is limited evidence regarding the impact of the CoLC on perceived health status and wellbeing across all groups, and whether specific groups of people (e.g., a specific age group, gender or socioeconomic status) are particularly affected by the ongoing economic crisis. There is also contrasting and limited evidence regarding the impact of the CoLC on individuals according to gender, their location (rural vs. urban), and age.

The aim of this study was to further understand the nuanced differences in the perceived impact on health and wellbeing of the CoLC in four European countries, (The U.K., Italy, Germany and Sweden). These countries were selected for comparison as they were all high-income, but demonstrated variability in the degrees of social safety nets & welfare provided, and had variably reported the impact of the CoLC [21, 22]. Within this aim, there were two research objectives:

1. To explore associations between the impact of the CoLC and participants’ reported life and health.
2. To explore the impact of the CoLC on reported life and health in four different yet comparable countries within Europe.

## Methods

### Study design

This study was designed as a cross-sectional survey to be conducted by the polling company YouGov [23] in collaboration with the research team at Imperial College London. YouGov has assembled nationally representative panels of adults who complete surveys periodically. This survey study is reported in line with the CROSS guidelines [24].

### Survey development

The complete survey was created by the study team at Imperial College London (ICL), building upon existing literature. It included questions relating to participants reported health and the impact of the CoLC on their life, access to healthcare services and other health-related activities (Supplementary File 1). Two of the questions within this survey related to the study research objectives and are reported on within this paper. The survey was iterated through collaboration by ICL and YouGov, with YouGov providing advice on framing the survey questions, and content driven by the research team at ICL.

### Participant characteristics

Participants were eligible to take part in this survey if they were over 18, residing in either Italy, Germany, the U.K. or Sweden, and were already signed up to YouGov’s polling system. The sample size for the survey was determined by YouGov, with an aim of 2000 participants from each country.

To ensure results from each participating country’s survey were nationally representative, YouGov assigns a post-stratification weight to each respondent to account for differences between the demographic composition of a country and that of the survey respondents. This method is adapted to each country by using different metrics to appropriately weight each participant. For example, in the U.K., the key demographics used are gender, age, education, social grade and religion. Respondents belonging to an under-represented demographic are weighted higher than those from over-represented demographics [25]. This process ensures that surveys are nationally representative by taking into account participants’ gender, education, race, level of education, social class and age [26].

### Data collection and variables

Surveys were administered through YouGov’s online portal, using their “active sampling” approach, in which only specifically selected individuals already signed up to the platform are invited to complete the survey. Participants completed the surveys between 17th March 2023 and 30th March 2023. The survey was conducted once per country. YouGov ensures that each participant is only able to respond to the survey once, avoiding multiple participation in the survey. As per standard practice by YouGov, the survey remains open on their polling platform until the desired sample size has been achieved.

Participants were shown a participation information sheet prior to commencing the survey, (translated as appropriate) and were asked to provide written informed consent. As participants were known to YouGov, the initial data collected by the polling platform was not anonymous. Pseudonymised data was transferred to the research team at ICL for analysis.

The survey collected data on participant demographics (gender, ethnicity, socio-economic status, location (e.g., urban vs. rural). It captured data on participants’ perceived overall health status.

The survey contained several questions pertaining to the impact of the CoLC, however two questions housed within the survey were the focus of this study as they addressed the two research objectives: *“Thinking in general*,* to what extent*,* if at all*,* has the cost of living crisis had a positive or negative impact on your life?”* and *“Thinking specifically about your health*,* to what extent*,* if at all*,* has the cost of living crisis had a positive or negative impact on you?”*.

YouGov also collects certain demographic data such as participants’ age, gender, ethnicity and location (urban, rural, mixed) in their country. Participants reported income was also collected as a separate question in the questionnaire.

### Statistical analysis

To investigate which variables were associated with participant’s reported impact of the CoLC on their life and health, logistic regression models were created for each country as well an overall model per question that included the data for all countries simultaneously. Two separate models were created using answers from the survey questions ‘Thinking in general, to what extent, if at all, has the cost of living crisis had a positive or negative impact on your life?’ and ‘Thinking specifically about your health, to what extent, if at all, has the cost of living crisis had positive or negative impact on you?’ as independent variables. For each model, negative answers (‘Major negative impact’, ‘Moderate negative impact’, ‘Minor negative impact’) were coded as ‘Negative’, and all others (‘No impact’, ‘Minor positive impact’, ‘Moderate positive impact’, ‘Major positive impact’) as ‘Positive/No change’. The covariates in our models were age groups (18–35, 36–64, 65+), gender (male, female), income quintile ( 1 to 5, derived from participants reported income, quintile 1 indicates those with the lowest income, quintile 5 those with the highest income), location (urban, mixed, rural, no data provided) and reported good health in the past 12 months (derived from the survey question ‘Thinking in general about the last 12 months, how would you classify your overall health?’, answers ‘Very poor’, ‘Poor’ and ‘Prefer not to say’ were coded as ‘No’, answers ‘Fair’, ‘Good’ and ‘Very good’ were coded as ‘Yes’). Overall models that included data from all countries also included an additional variable denoting each country.

All analyses were conducted using R (V 4.2.1) and RStudio (V 2023.06.1 + 524). Summary demographic tables were created by calculating the total number of participants for each category as well as the percentage of the total they represent. Logistic regression models were created using base R function *glm.* Overall models also included the participants country as a covariate. Samples in the model were weighted based on the weights provided by YouGov. All statistical analysis used a confidence level of 0.05. Models were inspected for violations of model assumptions using the *performance* package (V 0.11.0). All models were satisfactory and showed low levels multicollinearity as all variables had a variance inflation factor bellow 1.15.

## Results

### Respondent characteristics

A total of 8,152 unique individuals responded to the survey and were included in the analysis (Table 1), with participants recruited equally from each participating country: Italy (*n* = 2,036), Germany (*n* = 2,027), Sweden (*n* = 2,017) and the United Kingdom (*n* = 2,027). The survey was closed by YouGov when the pre-determined sample size had been achieved.

Individuals aged 36–64 were the most prevalent group (53.2%), compared to those 18–35 (26.4%) and 65+ (20.3%). There was a similar gender split across all countries (50.9% female, 49.1% male). Income quintiles were calculated based on the reported income bands per country, however, 19.6% of the overall study population did not report their income. In terms of location, most individuals resided in an urban setting (62.3%), compared to rural (22.2%) and mixed (14.6%). The UK was the only country in which some individuals (76, 3.7%) did not report their location. Finally, across all countries, most individuals (82.3%) reported having good health in the 12 months prior to the survey.

**Table 1:** Number and proportion of survey participants per country and variable included in the analysis

		UK (*n* = 2,072)	Italy (*n* = 2,036)	Germany (*n* = 2,027)	Sweden (*n* = 2,017)	All countries (*n* = 8,152)
Age	18–35	586 (28.3%)	479 (23.5%)	520 (25.7%)	571 (28.3%)	2,156 (26.4%)
	36–64	963 (46.5%)	1248 (61.3%)	1149 (56.7%)	979 (48.5%)	4,339 (53.2%)
	65+	523 (25.2%)	309 (15.2%)	358 (17.7%)	467 (23.2%)	1,657 (20.3%)
Gender	Female	1062 (51.3%)	1054 (51.8%)	1045 (51.6%)	991 (49.1%)	4,152 (50.9%)
Income quintile	1	323 (15.6%)	327 (16.1%)	329 (16.2%)	333 (16.5%)	1,312 (16.1%)
	2	323 (15.6%)	327 (16.1%)	329 (16.2%)	333 (16.5%)	1,312 (16.1%)
	3	323 (15.6%)	327 (16.1%)	329 (16.2%)	333 (16.5%)	1,312 (16.1%)
	4	323 (15.6%)	327 (16.1%)	329 (16.2%)	332 (16.5%)	1,311 (16.1%)
	5	323 (15.6%)	327 (16.1%)	328 (16.2%)	332 (16.5%)	1,310 (16.1%)
	No data provided	457 (22.1%)	401 (19.7%)	383 (18.9%)	354 (17.6%)	1,595 (19.6%)
Location	Urban	1,570 (75.8%)	1,220 (59.9%)	992 (48.9%)	1,296 (64.3%)	5,078 (62.3%)
	Rural	246 (11.9%)	358 (17.6%)	718 (35.4%)	487 (24.1%)	1,809 (22.2%)
	Mixed	180 (8.7%)	458 (22.5%)	317 (15.6%)	234 (11.6%)	1,189 (14.6%)
	No data provided	76 (3.7%)	0 (0%)	0 (0%)	0 (0%)	76 (0.9%)
Previous good health	Yes	1,682 (81.2%)	1,778 (87.3%)	1,681 (82.9%)	1,569 (77.8%)	6,710 (82.3%)

### Research objective 1: Associations between the impact of the CoLC and participants’ reported life and health

When investigating the impact of the CoLC on participants’ reported life across all four participating countries (Table 2, survey question: *“Thinking in general*,* to what extent*,* if at all*,* has the cost of living crisis had a positive or negative impact on your life?“*), individuals aged 36–64 were 42% (*p* < 0.001) more likely to report a negative impact on their life compared to those aged 18–36, while those aged 65 + did not show a significant difference. Females were 12% (*p* = 0.041) more likely than males to report a negative impact on their life. With relation to participants’ income, when compared to those in quintile 3 of earnings, those in quintile 1 were 37% more likely (*p* = 0.002) to report a negative impact, while those in quintile 5 were 38% (*p* < 0.001) less likely. Those that did not report their earnings were 17% less likely (*p* = 0.038) to report a negative impact compared to those in income quintile 3. Participants that reported not having good health in the previous twelve months were 85% more likely (*p* < 0.001) to report a negative impact on their life compared to those who perceived themselves to be in good health. Finally, compared to UK residents, those in Italy were 59% more likely (*p* < 0.001) to report a negative impact of the CoLC on their life, while those in Germany and Sweden were 19% (*p* = 0.008) and 35% (*p* < 0.001) less likely to report a negative impact on their life. There were no significant differences identified based on participants’ location within their country (rural/urban/mixed).

When analysing the impact of the CoLC on participants’ health (Table 2, survey question: *“Thinking specifically about your health*,* to what extent*,* if at all*,* has the cost of living crisis had a positive or negative impact on you?“*) participants aged 36–64 were 14% more likely (*p* = 0.020) to report a negative impact on their health compared to those aged 18–35. Participants aged 65 + were 34% less likely (*p* < 0.001) to report a negative impact on their health when again compared to those aged 18–35. Females were 10% more likely (*p* = 0.037) to report a negative impact on their health. Individuals in lower income quintiles 1 and 2 were 48% (*p* < 0.001) and 26% (*p* = 0.005) more likely, respectively, to report a negative impact on their health compared to those in income quintile 3. Participants in income quintile 4, 5 and those that did not report their income where 15% (*p* = 0.047), 41% (*p* < 0.001) and 22% (*p* = 0.002) less likely than those in income quintile 3, respectively, to report a negative impact on their health. Those that reported having poor health prior to the survey were 3.16 times more likely to report a negative impact on their health compared to those that reported good health. Finally, compared to participants in the UK, those in Italy were 32% (*p* < 0.001), more likely to report a negative impact, while those in Germany (24%, *p* = 0.029) and Sweden (41%, *p* < 0.001) were less likely to report a negative impact. There were no significant differences identified based on participants’ location (rural/urban/mixed).

**Table 2:** Results from multivariate logistic regression analysis modelling the likelihood of reporting a negative impact on either participants life of health as a consequence of the cost of living crisis. Values are shown as odds ratios and 95% confidence intervals. ‘***’ *p* < 0.001, ‘**’ *p* < 0.01, ‘*’ *p* < 0.05

Variable	Category	Impact on life	Impact on health
Age (Ref. category: 18–35)	36–64	1.42 (1.26–1.61) ***	1.14 (1.02–1.27) *
	65+	0.98 (0.84–1.14)	0.68 (0.59–0.78) ***
Gender (Ref. category: Male)	Female	1.12 (1-1.24) *	1.1 (1.01–1.21) *
Location (Ref. category: Urban)	Rural	1.05 (0.92–1.2)	0.94 (0.84–1.06)
	Mixed	1.09 (0.93–1.29)	1.06 (0.93–1.21)
	No data provided	1.38 (0.76–2.72)	1.09 (0.67–1.78)
Income quintile (Ref. category: Quintile 3)	Quintile 1	1.37 (1.12–1.67) *	1.48 (1.26–1.73) ***
	Quintile 2	1.17 (0.97–1.42)	1.26 (1.07–1.47) *
	Quintile 4	0.98 (0.81–1.18)	0.85 (0.73-1) *
	Quintile 5	0.62 (0.52–0.74) ***	0.59 (0.5–0.69) ***
	No data provided	0.83 (0.69–0.99) *	0.78 (0.67–0.91) *
Reported previous good health (Ref. category: Yes)	No	1.85 (1.58–2.17) ***	3.16 (2.78–3.59) ***
Country (Ref. category: UK)	Italy	1.59 (1.35–1.88) ***	1.32 (1.15–1.5) ***
	Germany	0.81 (0.7–0.95) *	0.86 (0.75–0.98) *
	Sweden	0.65 (0.56–0.75) ***	0.69 (0.6–0.78) ***

### Research objective 2: The impact of the CoLC in four different countries within Europe

### The impact of the CoLC on participant’s reported life

Looking at the perceived impact of the CoLC on participants life in each country (Table 3) our models indicated that age had different impacts across countries. In the UK, Germany, and Italy, participants aged 36–64 were 60% (*p* < 0.001), 64% (*p* < 0.001) and 53% (*p* < 0.0001) more likely to report negative impacts on their life respectively compared to those aged 18–35. In Germany and Italy, those aged 65 + were 55% (*p* = 0.007) and 185% (*p* < 0.001) more likely to report negative impacts on their life compared to those aged 18–35, while in Sweden, participants aged 65 + were 44% (*p* < 0.001) less likely.

In three countries (UK, Germany and Italy) females were 30% (*p* = 0.018) 32% (*p* = 0.008) and 60% (*p* < 0.001) more likely than males to report a negative impact on their life respectively, while those in Sweden were 29% less likely to report a negative impact (*p* < 0.001) than males.

Overall, the CoLC was perceived by participants to have had a more negative impact on those in the lowest income bracket (quintile 1). In Germany, individuals in income quintile 1 were 71% (*p* = 0.009) more likely to report a negative impact of the CoLC than those in the higher income bracket of quintile 3. This was also seen in Sweden, with participants in income quintile 1 56% more likely (*p* = 0.017) to report a negative impact than those in quintile 3. Meanwhile, survey respondents in Germany, Italy and Sweden in income quintile 5 (the highest income bracket) were 43% (*p* = 0.001), 44% (*p* = 0.006) and 35% (*p* = 0.011) less likely respectively to report a negative impact on their life compared to those in quintile 3.

Finally, participants who reported poorer health in the last 12 months were more likely in all countries (UK 91% *p* < 0.001, Germany 51% *p* < 0.0001, Italy 82% *p* < 0.0001, Sweden 105% *p* < 0.0001) to report negative impacts on their life compared to those with good health.

**Table 3:** Results from multivariate logistic regression analysis modelling the likelihood of reporting a negative impact on participants life as a consequence of the cost of living crisis per country. Values are shown as odds ratios and 95% confidence intervals. ‘***’ *p* < 0.001, ‘**’ *p* < 0.01, ‘*’ *p* < 0.05

Variable	Category	UK	Germany	Italy	Sweden
Age (Ref. category: 18–35)	36–64	1.6 (1.24–2.06) ***	1.64 (1.3–2.08) ***	1.53 (1.15–2.03) *	1.13 (0.89–1.43)
	65+	0.8 (0.6–1.05)	1.55 (1.13–2.14) *	2.85 (1.83–4.57) ***	0.58 (0.45–0.76) ***
Gender (Ref. category: Male)	Female	1.3 (1.05–1.61) *	1.32 (1.07–1.63) *	1.6 (1.24–2.07) ***	0.71 (0.58–0.87) ***
Location (Ref. category: Urban)	Rural	0.92 (0.67–1.28)	1.16 (0.92–1.46)	0.92 (0.66–1.29)	1.03 (0.81–1.3)
	Mixed	1.06 (0.73–1.58)	1.08 (0.8–1.46)	1.21 (0.88–1.69)	1.09 (0.8–1.51)
	No data provided	1.34 (0.73–2.64)			
Income quintile (Ref. category: 3)	Quintile 1	1.34 (0.9–1.99)	1.71 (1.15–2.56) *	0.73 (0.46–1.15)	1.56 (1.08–2.26) *
	Quintile 2	1.1 (0.76–1.61)	1.23 (0.84–1.79)	1.07 (0.65–1.75)	1.18 (0.83–1.67)
	Quintile 4	1.39 (0.94–2.07)	0.85 (0.6–1.22)	0.7 (0.44–1.11)	1.02 (0.72–1.43)
	Quintile 5	0.72 (0.5–1.04)	0.57 (0.4–0.8) *	0.54 (0.35–0.84) *	0.65 (0.47–0.91) *
	No data provided	0.94 (0.67–1.33)	0.8 (0.57–1.13)	0.66 (0.42–1.02)	0.79 (0.56–1.1)
Reported previous good health (Ref. category: Yes)	No	1.91 (1.4–2.64) ***	1.51 (1.12–2.06) *	1.82 (1.17–2.97) *	2.05 (1.57–2.7) ***

### The impact of the CoLC on participant’s reported health

Looking at the impact of the CoLC on participants’ health within each country (Table 4), participants aged 36–64 were 43% (*p* = 0.001) and 49% (*p* < 0.001) more likely to report negative impacts on their life compared to those aged 18–35 in the UK and Italy, respectively. In Italy, those aged 65 + were 77% more likely (*p* < 0.001) to report a negative impact, while those in UK, Germany and Sweden were 37% (*p* < 0.001), 25% (*p* = 0.035) and 67% (*p* < 0.001) less likely (compared to those aged 18–35).

Females were 42% (*p* < 0.001) and 23% (*p* = 0.001) more likely to report negative impacts on their life compared to males in Germany and Italy, while they were 29% less likely (*p* < 0.001) in Sweden.

Rural residents in the UK were 28% (*p* = 0.024) less likely than those in urban areas to report negative impacts on their life.

When comparing participants by income, those in quintile 1 in Germany and Sweden were 37% (*p* = 0.002) and 33% (*p* = 0.002) more likely to report negative impacts on their health. In Sweden, those in quintile 2 also reported a 56% increase in likelihood. On the other hand, individuals in quintile 5 were less likely to report negative impacts on their life compared to those in quintile 3 (UK: 34%, *p* = 0.011; Germany: 18%, *p* = 0.016; Italy: 54%, *p* < 0.001; Sweden: 44%, *p* = 0.002). UK participants who did not report their income were also 34% (*p* = 0.011) less likely to report a negative impact to their health.

Finally, participants from all countries who reported poor health in the previous twelve months were more likely to report a negative effect on their health than those with good health (UK: 3.48 times, *p* < 0.001; Germany: 2.34 times, *p* < 0.001; Italy: 3.58 times, *p* < 0.001; Sweden: 3.52 times, *p* < 0.001).

**Table 4:** Results from multivariate logistic regression analysis modelling the likelihood of reporting a negative impact on participants health as a consequence of the cost of living crisis per country. Values are shown as odds ratios and 95% confidence intervals. ‘***’ *p* < 0.001, ‘**’ *p* < 0.01, ‘*’ *p* < 0.05

Variable	Category	UK	Germany	Italy	Sweden
Age (Ref. category: 18–35)	36–64	1.43 (1.16–1.78) *	1.01 (0.81–1.25)	1.49 (1.19–1.86) ***	0.86 (0.69–1.07)
	65+	0.63 (0.49–0.82) ***	0.74 (0.55–0.98) *	1.77 (1.31–2.4) ***	0.33 (0.25–0.44) ***
Gender (Ref. category: Male)	Female	1.09 (0.91–1.31)	1.42 (1.19–1.71) ***	1.36 (1.13–1.63) *	0.71 (0.58–0.86) ***
Location (Ref. category: Urban)	Rural	0.72 (0.54–0.96) *	1 (0.82–1.23)	0.85 (0.66–1.08)	1.1 (0.88–1.38)
	Mixed	0.82 (0.59–1.13)	1.01 (0.77–1.31)	1.16 (0.92–1.46)	1.22 (0.9–1.65)
	No data provided	1.02 (0.62–1.67)			
Income quintile (Ref. category: 3)	Quintile 1	1.38 (0.99–1.92)	1.63 (1.19–2.24) *	1.21 (0.87–1.67)	1.67 (1.21–2.33) *
	Quintile 2	1.03 (0.75–1.43)	1.36 (0.99–1.86)	1.2 (0.87–1.66)	1.44 (1.04-2.00) *
	Quintile 4	0.74 (0.53–1.01)	0.97 (0.7–1.33)	0.78 (0.57–1.07)	0.93 (0.67–1.29)
	Quintile 5	0.55 (0.4–0.77) ***	0.67 (0.49–0.93) *	0.56 (0.4–0.76) ***	0.58 (0.41–0.82) *
	No data provided	0.68 (0.5–0.92) *	0.82 (0.6–1.11)	0.8 (0.59–1.09)	0.79 (0.57–1.1)
Reported previous good health (Ref. category: Yes)	No	3.48 (2.72–4.5) ***	2.34 (1.83–3.01) ***	3.58 (2.6–5.01) ***	3.52 (2.79–4.46) ***

## Discussion

### Key findings

This survey demonstrates the perceived negative impact on life and health of the CoLC, and highlights population subgroups who are more likely to be disproportionately affected. We add to the existing body of literature by highlighting that across all four European countries, older individuals, females, those from lower income families and those reporting poor health in the previous 12 months were all significantly more likely to report a negative impact on their life and health. Interestingly, participant location (e.g., urban vs. rural) had no influence on the perceived impact. This study uniquely highlights the nuanced differences in the impact of a CoLC as demonstrated in four European countries, with differences reported according to age, gender and participant location.

### Interpretations

The four European countries included in this survey were selected as they were perceived to all be high income and have broadly comparable populations. However, within this some significant differences in the perceived impact of the CoLC on life and health were demonstrated in the different countries. A lower impact of the CoLC was felt in Germany and Sweden than the UK, with Italy reporting the highest perceived impact.

Overall, for the entire study population, individuals aged 36–64 were more likely to report a perceived impact on their life and health when compared to older and younger participants – this may be as a consequence of having more financial responsibilities / dependents than other age groups. Those aged 18–35 were less likely to report an impact on their health than the two older age group cohorts – possibly a reflection of reduced co-morbidities in this younger cohort.

There was evidence of a disparity in the impact of the CoLC according to gender – overall in our survey females were more significantly likely to report a negative impact of the CoLC on their life and health. This may be a reflection of the fact females are more likely to be unpaid primary care givers, in part-time work or have lower state pensions [27].

Unsurprisingly, those with a lower income or pre-existing poor health were more likely to report a negative impact of the CoLC on their life and health. This is likely to reflect the fact that a larger proportion of the income for these participants is apportioned to essentials such as food and heating bills, and therefore they may be disproportionately affected by rising prices in these areas, and these households have less in the way of savings to buffer such an increase. Our study findings are in line with other research reporting that those from lower incomes or lower socioeconomic demographics are more likely to be affected by a CoLC [28].

Our study found that there was no significant difference in the perceived impact of the CoLC on health or life based upon the location of the participant (be that rural or urban), when analysed across all countries. Of note, the UK demonstrated that those living in rural areas were less likely to perceive a negative impact. This contrasts a recent study by the Rural Services Network which found that those in rural areas were more likely to be disproportionately affected as a result of higher living costs (with homes being more difficult to heat) and lower incomes [29], (this in itself contrasting other literature stating the converse [30]).

Interestingly, when analysed in more detail according to individual countries, some differences in impact according to age are unveiled. In Sweden, older adults are less likely to report a negative impact of the CoLC for both health and life outcomes. In Italy, this age group were more likely to report a negative outcome.

Further disparity emerges in the impact of the CoLC when other population variables are analysed according to country – with Sweden showing that females reporting being *less* likely to have perceived negative impacts on life and health – different to the other three countries. This may be attributable to an increased ethos of gender equality in Scandinavian countries [31].

### Limitations

There are several limitations associated with this study. Firstly, it only incorporated four countries, all of which had similar baseline socioeconomic demographics. Whilst differences in these countries were highlighted, other countries with different challenges may respond differently to a CoLC. It is also possible that the survey may not have captured other systemic factors that might have had an impact as mediators / confounders such as the cost of accessing health care or the provision of social care.

Our study did not account for differences in welfare or compensatory measures. To help mitigate the impact of the CoLC, the four countries in our study all brought in new support packages. Italy reduced taxation on energy goods in early 2022 and granted subsidies to poorer households [4]. Sweden also brought in electricity subsidies for those who needed them and boosted housing allowances [32]. Germany introduced a price cap on basic energy consumption, provided energy vouchers and increased the number of individuals who received housing benefits [33], and the UK government implemented a £78 billion package to support households, including an energy price guarantee and a monetary discount on energy bills [34]. There are similarities and differences in these welfare policies, and the differential impact on mitigating the CoLC in each country is an interesting topic for future work. In spite of these differing policies, which may well have reduced the effects of inflation, our study does still demonstrate a disproportionate impact of the crisis on certain population subgroups, which is seen in all four countries. As such, there is still work to do in developing equitable responses to such events.

The survey participants were limited to individuals already registered on the YouGov polling platform. Whilst this platform strives to have participants who reflect the demographics of their country, it is possible that this may have introduced bias into our study – those who are motivated to participate in such online surveys may be more or less affected by the crisis, and our conclusions should be interpreted with this in mind. A further limitation is that the study team relied on YouGov to weight the participants (in order to create a more representative participant demographic), rather than truly sampling the population. Whilst YouGov adapts this weighting according to the appropriate key demographic variables for each included country, we acknowledge that there is still a risk that not all demographics may have been represented accurately and this should be taken into account when interpreting the results.

It is also possible that the language used in our survey – “cost of living **crisis**”, may have introduced a negative bias in terms of how participants responded, given the negative connotations associated with the word crisis. However, we wanted to use a term that was familiar to individuals and allowed them to describe the impact of the CoLC on the things that they felt were important to them.

### Generalisability

Whilst this survey highlighted differences in the impact of the CoLC in four participating countries, there were consistencies demonstrated when evaluating the impact of the CoLC. As such, these findings may be generalisable across comparable countries (in relation to income level, or those with similar systemic structures). Given that in all four countries, those in the age group 36–64, in lower incomes, with poorer health all reported a greater impact of the CoLC, it is reasonable to expect that these findings would be similar in comparable countries. The study findings also demonstrate that external “shocks” such as a CoLC will impact different groups within society in different manners – learning which can be applied when developing preparedness strategies for future similar events.

### Opportunities for future work

This study provided data on participants self-reported perception of the impact of the CoLC. It would be of value to repeat this study with a more objective measure of the impact on health and life – for example using quality of life metrics. It would also be beneficial to expand this survey to other countries affected by the CoLC globally – to identify the impacts and disparities across nations, build further understanding as to why these occur and develop strategies to mitigate the negative impact. It would also be interesting to research in depth why some countries (in this case Germany and in particular Sweden) appear to be more resilient to events such as a CoLC – evaluating their key policies and welfare mechanisms and identifying opportunities to implement these in other, more affected areas.

## Conclusions

The results of this survey highlight the subtly different impact of the CoLC on different participant demographics as seen in four countries in Europe. It is likely that there are many contributing factors to the aetiology of this difference in impact, which will need further research to understand. However, demonstrating this difference in impact should mean that strategies to mitigate the impact of the CoLC can be more targeted to those who are most in need, with countries learning from each other.

### Electronic supplementary material

Below is the link to the electronic supplementary material.

Supplementary Material 1

## Data Availability

The anonymised datasets used and analysed during the current study are available from the corresponding author on reasonable request.
