# Supplementary material for: The cost of living crisis – how does it impact the health and life of individuals? A survey exploring perceptions in Italy, Germany, Sweden and the United Kingdom
Source: BMC Public Health. 2024 Jul 9;24:1831. doi: 10.1186/s12889-024-19330-y (PMC11234749; doi:10.1186/s12889-024-19330-y)
Supplement: Supplementary file 1 — Supplementary Material 1 [file 12889_2024_19330_MOESM1_ESM.docx]

**Supplementary File 1: YouGov Survey Questions**

**Impact of cost-of-living crisis on patient safety**

**PART I: Participant information**

In this section, we aim to learn more about your characteristics.

**1. Please select your gender**

- Female
- Male
- Other
- Prefer not to answer

**2. Please select your ethnicity:**

**[Categories]**

**3. How would you describe your current socio-economic status:**

- Low
- Middle-low
- Middle
- Middle-high
- High

**4. How would you describe the setting in which you live:**

- Urban
- Rural
- Mixed

**5. In general this year, how would you classify your overall health status?**

- Likert scale [Very poor / poor / fair / good / excellent], 5 categories

**PART II: Impact on life and health**

In this section, we aim to learn more about how the cost-of-living crisis has impacted your life and health. By cost-of-living crisis we mean the impact high inflation on essential commodities, such as food and energy, is having on standards of living.

**6. Has the cost-of-living crisis had a negative impact on your life, overall?**

- No impact
- Minor impact
- Moderate impact
- Major impact

**7. Has the cost-of-living crisis had a negative impact on your health, considering both direct and indirect impacts?**

- No impact
- Minor impact
- Moderate impact
- Major impact

**8. How has the cost-of-living crisis negatively impacted the different aspects of your health and healthcare listed below?**

|  | **No impact** | **Minor impact** | **Moderate impact** | **Major impact** |
| --- | --- | --- | --- | --- |
| **Lifestyle habits (I.e., diet, exercise)** |  |  |  |  |
| **Mental health** |  |  |  |  |
| **Taking your medication** |  |  |  |  |
| **Getting vaccinated** |  |  |  |  |
| **Getting cancer screening tests (e.g. colonoscopy, mammography)** |  |  |  |  |
| **Managing your chronic disease (e.g. diabetes, asthma)** |  |  |  |  |
| **Seeing the primary care (GP)** |  |  |  |  |
| **Going to urgent/emergency care** |  |  |  |  |
| **Ease of getting an appointment with my GP/family doctor** |  |  |  |  |
| **Ease of getting care from a specialist or hospital (not my GP/family doctor)** |  |  |  |  |
| **Ease of getting care at the emergency department** |  |  |  |  |

**9. In case there are other additional aspects in which the cost-of-living crisis has negatively impacted your health, please explain below.**

- [free-text answer]

**10. Has the cost-of-living crisis changed your preferences about your preferred mode of care delivery?**

- No change, I have always preferred face-to-face care
- No change, I have always preferred virtual consultations (i.e., telephone or video) whenever appropriate
- I have changed my preference to face-to-face care
- I have changed my preference to virtual consultations (i.e., telephone or video), whenever appropriate
